# An update on the bridging factors connecting autophagy and Nrf2 antioxidant pathway

**DOI:** 10.3389/fcell.2023.1232241

**Published:** 2023-08-09

**Authors:** Baike Ning, Shuqi Hang, Wenhe Zhang, Caiwen Mao, Dan Li

**Affiliations:** ^1^ Collaborative Innovation Center of Yangtze River Delta Region Green Pharmaceuticals, College of Pharmaceutical Sciences, Zhejiang University of Technology, Hangzhou, China; ^2^ Department of Molecular, Cellular, and Developmental Biology, University of Michigan, Ann Arbor, MI, United States

**Keywords:** bridging factors, autophagy, Nrf2-Keap1, TRIM16, SQSTM1, Sestrin2

## Abstract

Macroautophagy/autophagy is a lysosome-dependent catabolic pathway for the degradation of intracellular proteins and organelles. Autophagy dysfunction is related to many diseases, including lysosomal storage diseases, cancer, neurodegenerative diseases, cardiomyopathy, and chronic metabolic diseases, in which increased reactive oxygen species (ROS) levels are also observed. ROS can randomly oxidize proteins, lipids, and DNA, causing oxidative stress and damage. Cells have developed various antioxidant pathways to reduce excessive ROS and maintain redox homeostasis. Treatment targeting only one aspect of diseases with autophagy dysfunction and oxidative stress shows very limited effects. Herein, identifying the bridging factors that can regulate both autophagy and antioxidant pathways is beneficial for dual-target therapies. This review intends to provide insights into the current identified bridging factors that connect autophagy and Nrf2 antioxidant pathway, as well as their tight interconnection with each other. These factors could be potential dual-purpose targets for the treatment of diseases implicated in both autophagy dysfunction and oxidative stress.

## 1 Introduction

Autophagy is a metabolic process that takes place in eukaryotic cells, which use lysosomes to degrade proteins and damaged organelles for self-eating and self-renewal (Wong et al., 2020). Autophagy dysfunction contributes to a variety of pathologies, including lysosomal storage diseases, neurodegenerative diseases, chronic metabolic diseases, and cancer (Klionsky et al., 2021). Of note, these diseases are usually accompanied by elevated levels of reactive oxygen species (ROS) (Giordano et al., 2014; Ryter et al., 2019; Wu W. et al., 2021).

ROS are highly reactive and diffusive molecules, and endogenous ROS mainly come from cellular respiration in mitochondria (Wang et al., 2021; Sies et al., 2022). In addition, various organelles (endoplasmic reticulum and peroxisome) and enzymes (NADPH oxidase and p450 cytochrome) also produce ROS during metabolic processes (Wang et al., 2021; Sies et al., 2022). ROS are regarded as a “double-edged sword.” Physiologically low levels of ROS serve as a signal, promoting cell viability and immune function and maintaining cell homeostasis (Sies and Jones, 2020; Sies et al., 2022). Conversely, excessive ROS oxidize and damage cellular components, leading to cellular death (Davalli et al., 2016), which is implicated in many diseases (Luo et al., 2020; Yang and Lian, 2020). To maintain ROS at low levels, cells are controlled by multiple antioxidant systems, including various non-enzymatic and enzymatic antioxidant pathways (Alkadi, 2020; Forman and Zhang, 2021), as well as several antioxidant transcription factors. Furthermore, these antioxidants have different subcellular localizations, therefore eliminating ROS in different compartments (Brieger et al., 2012).

Interestingly, ROS also interplay with autophagy (Gao et al., 2020). Autophagy can participate in the redox balance by clearing oxidative stress-damaged molecules and organelles (Yun et al., 2020). The Nrf2-Keap1-ARE pathway plays a central role in adaptive cellular redox response (Shaw and Chattopadhyay, 2020; Esteras and Abramov, 2022). Studies have reported that several proteins serve as bridges between the Nrf2 pathway and autophagy (Li et al., 2021a; Zhang et al., 2021). In this review, we focus on the bridging factors that establish networks between autophagy and the Nrf2 antioxidant pathway.

### 1.1 ROS, oxidative stress, and antioxidant pathways

**FIGURE 1:**
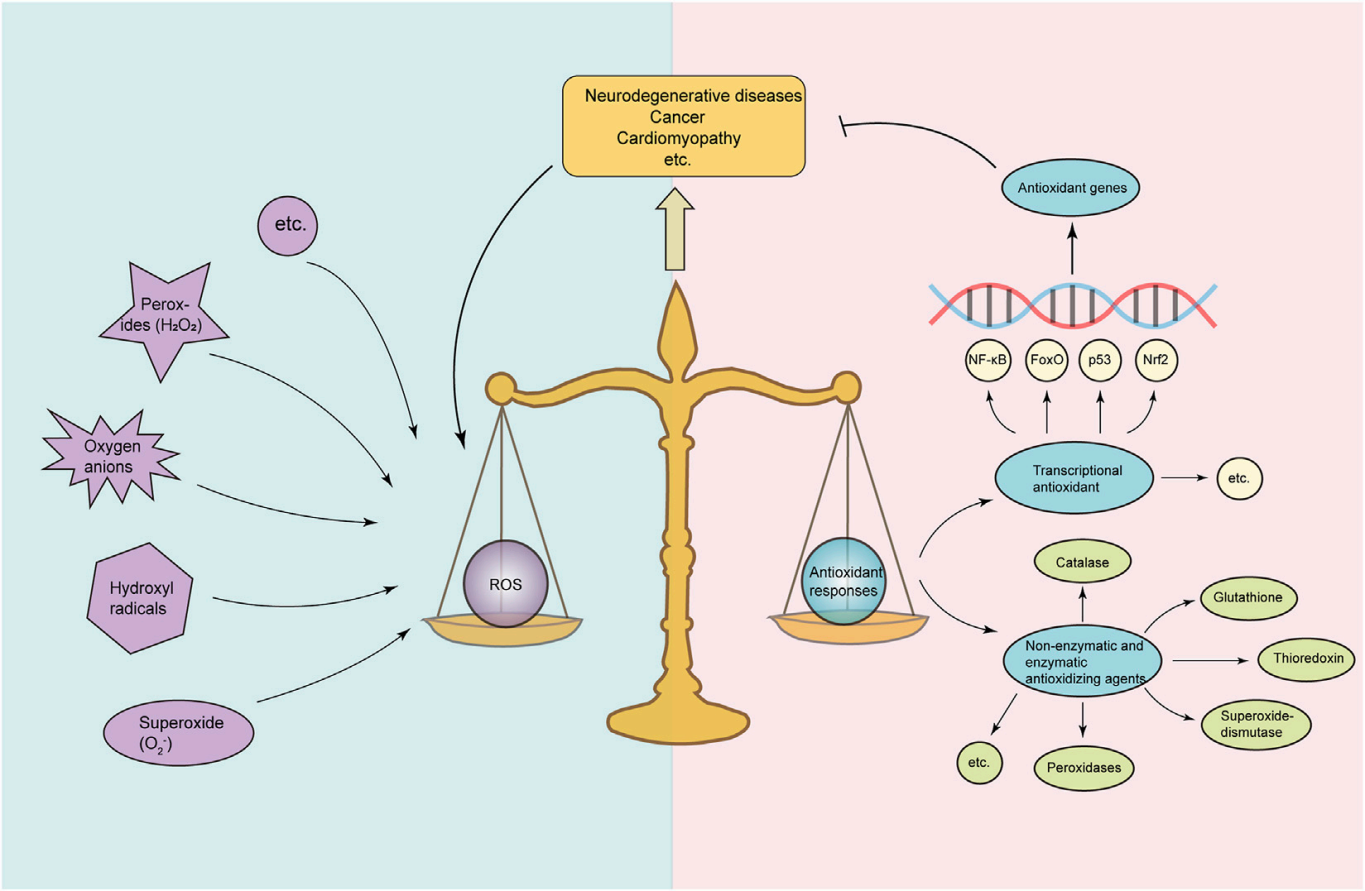
Balance between ROS and antioxidant responses. Under normal conditions, cells maintain redox homeostasis by balancing ROS levels and antioxidant activity. Cellular ROS including oxygen anions, superoxide (O_2_^−^), hydroxyl radicals, and hydrogen peroxides (H_2_O_2_) and antioxidant responses consisting of non-enzymatic and enzymatic antioxidant pathways (peroxidases, superoxide dismutase, thioredoxin, and glutathione) and transcriptional antioxidant pathways (including Nrf2, NF-κB, FoxO, and p53) form a balance. Under certain pathological conditions such as neurodegenerative diseases, cancer, and cardiomyopathy, excessive ROS accumulation overloads the antioxidant responses and breaks the cellular homeostasis, resulting in oxidative stress and mitochondrial dysfunction, contributing to the progression of diseases.

ROS define several highly reactive, short-lived molecules, which are formed during the oxygen reduction process (Shadel and Horvath, 2015). Intracellular ROS are mainly produced by the electron transport chain of mitochondria and NADPH oxidase (Lennicke and Cochemé, 2021). Other sources that generate ROS include the endoplasmic reticulum, peroxisome, and nucleus (Lennicke and Cochemé, 2021). Endogenous ROS generally contain superoxide (O_2_^−^), hydrogen peroxide (H_2_O_2_), hydroxyl radicals, and oxygen anions (Shadel and Horvath, 2015). Physiological ROS act as signaling molecules, regulating physiological and metabolic functions and promoting cell proliferation and innate immune response (Mittler, 2017; Forrester et al., 2018); conversely, pathological or exogenous stimulus can induce ROS production and accumulation (Zorov et al., 2014; Yuan et al., 2019). To maintain redox homeostasis, cells have developed diverse antioxidant pathways to counteract ROS (Figure 1). However, in response to pathological or exogenous stimuli, antioxidant pathways are overloaded with excessive ROS, leading to oxidative stress, which causes damage to lipids, proteins, and DNA (Sies, 2015; Forman and Zhang, 2021). Various non-enzymatic and enzymatic antioxidant pathways are involved in reducing ROS and preventing oxidative stress, including superoxide dismutases, thioredoxin, catalases, peroxidases, and glutathione (Elias et al., 2008; He et al., 2017). Furthermore, multiple transcriptional antioxidant pathways, i.e., Nrf2, NF-κB, FoxO, and p53, make an important contribution to anti-oxidation (Marinho et al., 2014) (Figure 1). In particular, the Nrf2-Keap1-ARE pathway plays a central role in the regulation of antioxidant enzyme genes and cytoprotective defense (Lu et al., 2016; Liu S. et al., 2022).

### 1.2 Nrf2-Keap1-ARE signaling pathway

Nuclear factor erythroid 2-related factor 2 (Nrf2) is a basic leucine zipper transcription factor belonging to the Cap ‘n’ Collar family. Under quiescent conditions, cytosolic Nrf2 directly binds to Kelch-like ECH-associated protein 1 (Keap1), a substrate adapter protein for the Cul3–E3–ligase complex, resulting in rapid degradation of Nrf2 (Ulasov et al., 2022). Keap1 is also a cysteine (Cys)-rich sensor of redox damage (Yamamoto et al., 2018). However, with exposure to stimuli such as oxidative/xenobiotic stress, Nrf2 escapes from Keap1-mediated repression and translocates to the nucleus, where it dimerizes with small musculoaponeurotic fibrosarcoma proteins (sMaf). The activated Nrf2-sMaf heterodimer binds to antioxidant response element (ARE) sequences in the promoter of a tandem of antioxidant genes, including heme oxygenase-1 (HMOX1), aldo-keto reductases, glutathione, and glutathione-S-transferase (Hayes and Dinkova-Kostova, 2014; Tonelli et al., 2018), to trigger their expression. Numerous studies have revealed the complexity and diversity of the Nrf2-Keap1 pathway in regulating biological processes, including cell proliferation, differentiation, anti-inflammation, and cytoprotection (Murakami and Motohashi, 2015; Yamamoto et al., 2018). Nrf2 has also been demonstrated to regulate mitochondrial biogenesis (Esteras and Abramov, 2022). Two key regulators of mitochondrial biogenesis, namely, proliferator-activated receptor gamma coactivator 1-α and nuclear respiratory factor 1, which are responsible for mitochondrial DNA transcription, are under the control of Nrf2 (Piantadosi et al., 2008; Baldelli et al., 2013; Merry and Ristow, 2016).

It is known that ROS also interact with autophagy. ROS could promote the formation of autophagy. Autophagy, in turn, could reduce oxidative damage by engulfing and degrading oxidized substances.

### 1.3 ROS and autophagy interplay

**FIGURE 2:**
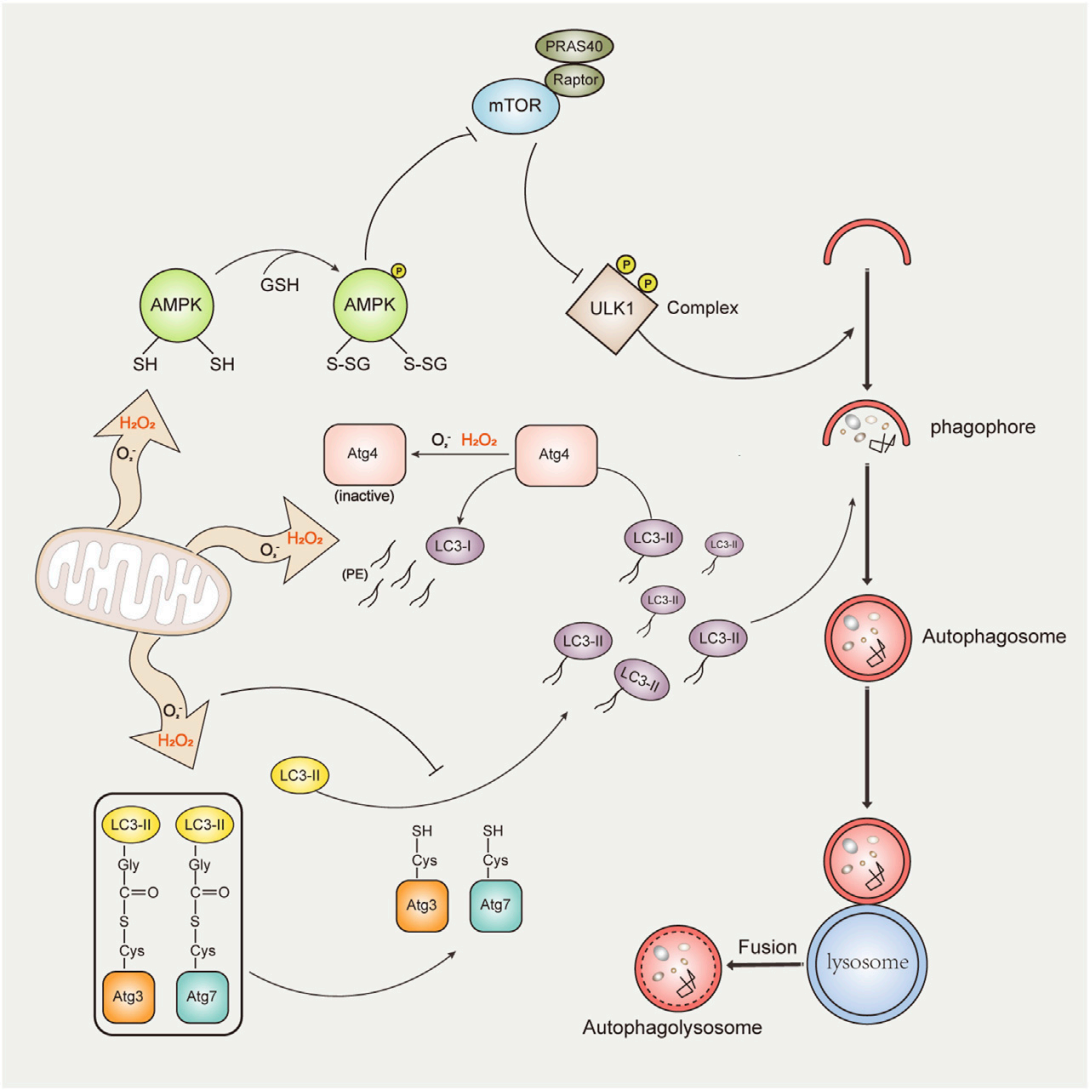
ROS and autophagy interplay. Mitochondria-released ROS (mainly H_2_O_2_ and O_2_^−^) regulate autophagy through three mechanisms: (1) ROS directly oxidize the cysteine residues of α and β subunits of AMPK, and activated AMPK inhibits the activity of the mTORC1 complex or phosphorylates the ULK1 complex, subsequently promoting autophagy (top). (2) Oxidation of Atg4 by ROS results in inactivation of LC3-PE deconjugation activity and accumulation of autophagic LC3-II isoforms, supporting autophagosome formation (middle). (3) The oxidation of catalytic thiols on Atg3 and Atg7 by ROS prevents the lipidation of LC3, thereby inhibiting autophagy (bottom). Autophagy, in turn, contributes to reduction of ROS and damaged organelles through pathways such as mitophagy. Damaged mitochondria are recruited into the autophagosome by binding with LC3. The mature autophagosome fuses with the lysosome to form the autolysosome, and the damaged mitochondria are subsequently degraded.

Autophagy is a catabolic process that is responsible for the degradation of intracellular proteins and damaged organelles in response to endogenous and exogenous stresses, including oxidative stress, endoplasmic reticulum stress, hypoxic stress, and nutrient and growth factor starvation (Galati et al., 2019). Promoting autophagy has been reported to have beneficial effects on longevity, anti-infection, and disease prevention, including diseases such as myocarditis, tumors, and neurodegenerative diseases (Petrache et al., 2008). Autophagy dysfunction has also been identified in many diseases, including Niemann–Pick type C (NPC) disease, atherosclerosis, and non-alcoholic steatohepatitis (Fischer et al., 2009). It is broadly recognized that excessive ROS can regulate autophagy (activation or inhibition) in various conditions (Gao et al., 2020; Ornatowski et al., 2020; Zhou et al., 2022). Autophagy, in turn, can also reduce oxidative damage by degrading oxidized substances (Li et al., 2015) (Figure 2). Thus, ROS and autophagy interact closely with each other. The process of autophagy is generally divided into the occurrence of phagophores, the formation of autophagosomes, the fusion of autophagosomes and lysosomes, and degradation. A variety of autophagy-related proteins (Atg) constantly regulate the whole process of autophagy (Levine and Kroemer, 2019).

Previous studies have identified that H_2_O_2_ and O_2_^−^ are major inducers of autophagy (Filomeni et al., 2015). H_2_O_2_ is relatively stable, long-lived, and highly selective to cysteine (Bienert and Chaumont, 2014). Instead, O_2_^−^ is unstable and easily converted to H_2_O_2_ spontaneously or catalyzed by enzymes (Fujii et al., 2022). In the initiation stage of autophagy, ROS (H_2_O_2_ and O_2_^−^) promote autophagy mainly via regulating the mechanistic target of rapamycin (mTOR). mTOR is a receptor of amino acids and ATP that plays a gating role in autophagy. ROS can activate AMP-activated protein kinase (AMPK) or inhibit protein kinase B (Zhao et al., 2017), which represses mTORC1 complex activity and enhances autophagy (Li et al., 2019; Guo et al., 2022). In the autophagosome formation stage, Atg4, an essential protease in autophagy, has been identified as a direct target of H_2_O_2_ (Scherz-Shouval et al., 2007). H_2_O_2_ directly oxidizes the Cys81 of Atg4 (Scherz-Shouval et al., 2007), thus leading to the inactivation of Atg4 and promoting the lipidation of light chain 3 (LC3, also the mammalian homolog of Atg8), thereby supporting the formation of autophagosomes (Pérez-Pérez et al., 2021). Interestingly, ROS-induced autophagy is primarily caused by O_2_^−^ during prolonged starvation (Chen et al., 2009). In contrast, H_2_O_2_ is produced immediately after starvation and regulates autophagy (Zhou et al., 2022). In addition, the crosstalk between autophagy and ROS also varies in different cell types. For example, in renal tubular cells, increased ROS can activate autophagy, leading to mitochondrial destruction and kidney damage (Duan et al., 2018). However, in tumor cells, ROS-induced autophagy activation can promote cell growth and cancer progression (Taucher et al., 2022).

Acute H_2_O_2_ can prevent LC3 lipidation, thereby inhibiting autophagy (Frudd et al., 2018). H_2_O_2_ oxidizes the cysteine residues of Atg3 and Atg7, thereby weakening their covalent interaction with LC3 (Frudd et al., 2018). Increased ROS can also inhibit autophagy by downregulating unc-51-like kinase 1 (ULK1) (Ci et al., 2014), an autophagy regulator. In addition, ROS also regulates autophagy through mitogen-activated protein kinase signaling pathways, including p38 kinase, extracellular signal-regulated kinase, and c-Jun amino-terminal kinase (Sui et al., 2014; Liu et al., 2015; Fan et al., 2020). Conversely, autophagy could regulate the levels of intracellular ROS. Selective autophagy, such as mitophagy and pexophagy, contributes to ROS reduction.

#### 1.3.1 Autophagy regulates ROS

Mitochondria are the primary sites of ROS production (approximately 90% of cellular ROS) (Balaban et al., 2005). Thus, mitochondria also contain a large number of antioxidants (proteins and enzymes), including glutathione, glutathione reductase, peroxiredoxin, and superoxide dismutase (Yang et al., 2020). Mitochondrial antioxidants reduce oxidative damage to mitochondria and maintain their homeostasis (Liang et al., 2012; Ristić et al., 2015; Kang et al., 2020). In pathological conditions, excessive ROS leads to oxidative stress, causing mitochondrial dysfunction and cellular damage (Tirichen et al., 2021). The process of specific and selective degradation of damaged mitochondria is called *mitophagy*. Mitophagy generally consists of two steps: the initiation of general autophagy and the priming of damaged mitochondria for autophagic recognition (Figure 2). Recently, studies have shown that ROS, as cellular signaling molecules, could trigger autophagosome formation and autophagic degradation (Filomeni et al., 2015; Roca-Agujetas et al., 2019).

In the process of mitophagy, mitophagy receptors or ubiquitin-autophagy adapters directly involve themselves in forming autophagosomes surrounding mitochondria and further degrading damaged mitochondria in autolysosomes, which are produced by the fusion of autophagosomes and lysosomes (Onishi et al., 2021). Recognition of receptor-mediated mitophagy depends on receptor proteins such as Atg32 in yeast (Okamoto et al., 2009) and several proteins in mammals, including NIP3-like protein X (Novak et al., 2010), Bcl-2 19-kDa interacting protein 3 (Zhu et al., 2013), FUN14 domain containing 1 (Liu et al., 2012), FK506-binding protein 8 (Bhujabal et al., 2017), and Bcl2-like 13 (Murakawa et al., 2015). These receptors contain a conserved key domain, the LC3-interacting region (LIR) domain, which can directly bind to Atg8/LC3 or other proteins in this family and then trigger mitophagy. In ubiquitin-mediated mitophagy, PTEN-induced putative kinase 1 accumulates on the outer membrane of damaged mitochondria, which promotes the E3 ubiquitin ligase-Parkin to be recruited to the surface of mitochondria. Subsequently, autophagy adapters (Sulkshane et al., 2021) (e.g., Atg 8 family, SQSTM1, a neighbor of BRCA1 gene 1, optineurin, Tax1-binding protein 1, and nuclear dot protein 52 kDa) directly recognize poly-ubiquitin chains on damaged mitochondria and then anchor them to phagocytic vesicles (Sulkshane et al., 2021).

#### 1.3.2 Pexophagy

Peroxisomes are small and highly dynamic organelles that are ubiquitous in eukaryotic cells and play a key role in cell metabolism and the regulation of redox homeostasis (Germain and Kim, 2020). *Pexophagy*, a process of selective autophagy for damaged peroxisomes, contributes to maintaining the intracellular redox balance (Germain and Kim, 2020). Under ROS stimulation, the peroxisome localization protein PEX5 (peroxisomal biogenesis factor 5) is phosphorylated at Ser141 by ataxia telangiectasia mutated kinase and subsequently ubiquitinated by E3 ligase (Zhang et al., 2015). SQSTM1 then recognizes the ubiquitinated PEX5 complex and triggers pexophagy (Zhang et al., 2015). In addition, PEX2 is identified as an E3 ubiquitin ligase during amino acid starvation (Sargent et al., 2016). PEX2 can ubiquitinate PEX5, thereby activating NBR1-dependent pexophagy (Sargent et al., 2016).

## 2 Bridging factors connecting autophagy and Nrf2-Keap1 pathway

Both autophagy dysfunction and oxidative stress have been implicated in many diseases. Treatment targeting one aspect shows limited effects. For example, N-acetylcysteine (NAC), a potent antioxidant, has been examined in clinical trials of NPC disease, which shows increased oxidative stress and dysfunctional autophagy (Fu et al., 2013; Wheeler and Sillence, 2020). Results show that NAC significantly reduces oxidative stress in NPC disease but has no effect on autophagic dysfunction-related cholesterol accumulation, thus failing in clinical trials (Fu et al., 2013). Of note, identifying the bridges that connect autophagy and antioxidant pathways could be extremely beneficial to novel dual-target therapies.

### 2.1 SQSTM1

SQSTM1 (sequestosome1, also called p62) is an autophagy adapter protein that participates in the degradation of protein aggregates and cytoplasmic bodies (Lin et al., 2013). Numerous studies have described the effects of SQSTM1 on a variety of diseases, such as Parkinson’s disease (PD) (Martins-Marques et al., 2015; Chu, 2019; Kumar et al., 2022). Under normal conditions, SQSTM1 recruits α-synuclein to form aggregates and eliminates them through autophagy. In contrast, in PD patients, dysfunctional SQSTM1 fails to recruit impaired α-synuclein, which accumulates in the central nervous system and aggravates PD progression (Shin et al., 2020). In addition, SQSTM1 can recognize ubiquitinated Parkin, a key protein of mitophagy, and disrupted SQSTM1 mediates mitophagy deficiency (Song et al., 2016), which is an important cause of PD.

SQSTM1 recruits ubiquitinated proteins and organelles to the autophagosome through its simultaneous interaction with multiple proteins, including protein kinase C and mTOR (Katsuragi et al., 2015). In autophagy-deficient cells, SQSTM1-induced Nrf2 activation is critical in tumor progression (Komatsu et al., 2010). SQSTM1 can directly bind to Keap1, disrupting Keap1-regulated Nrf2 degradation and promoting aberrant Nrf2-activation (Komatsu et al., 2010). The DPSTGE motif in the Keap1 interaction region (KIR) domain of SQSTM1 resembles the ETGE motif in Nrf2 (Komatsu et al., 2010) and illuminates the competitive binding of Keap1 between SQSTM1 and Nrf2. However, the binding ability of SQSTM1-KIR to Keap1 is much weaker than that of Nrf2-ETGE. Nevertheless, under exposure to ROS or other electrophilic stimuli, Ser351/349 within the DPSTGE motif in the KIR region will be phosphorylated, and consequently, the binding affinity of SQSTM1 to Keap1 can be drastically increased (Ichimura et al., 2013; Sánchez-Martín and Komatsu, 2018).

**FIGURE 3:**
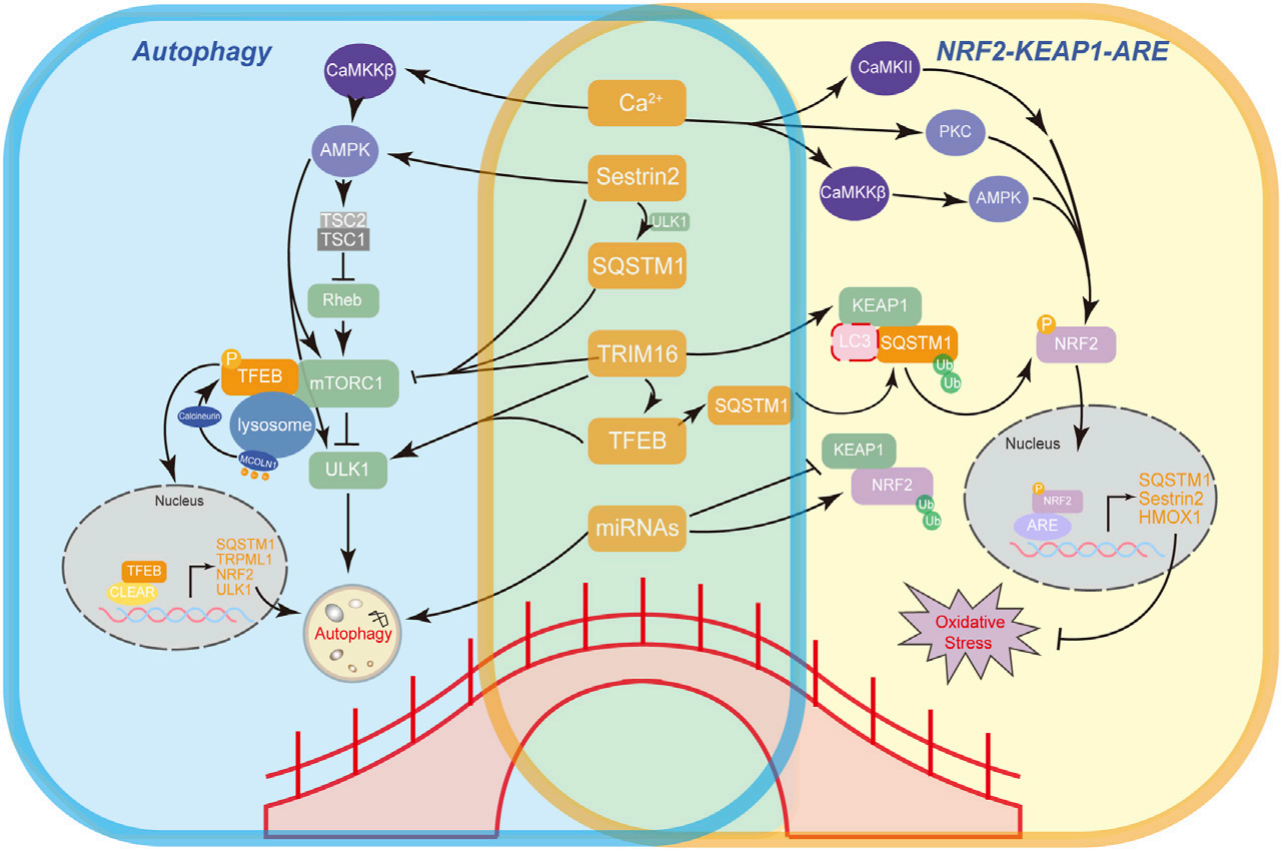
Bridging factors that connect autophagy and Nrf2 antioxidant pathway. A working model illustrates the mechanisms of bridging factors (SQSTM1, TFEB, Sestrin2, TRIM16, Ca^2+^, and miRNAs) connecting autophagy (left) and the main antioxidant Nrf2-Keap1-ARE pathway (right) and the feedback loops between these factors.

SQSTM1 can recruit Keap1 and LC3 to form the LC3–SQSTM1–Keap1 complex, which is then degraded by selective autophagy (Liao et al., 2019) (Figure 3). Nevertheless, the interaction between LC3, SQSTM1, and Keap1 still remains controversial, as the binding competition to SQSTM1 between Keap1 and LC3 can be attributed to the spatial adjacency of the LIR domain and KIR domain in SQSTM1 (Jain et al., 2010). Moreover, depleting SQSTM1 with siRNA leads to an almost twofold increase in the half-life of Keap1 (Copple et al., 2010), indicating that SQSTM1 could influence the basal level of Keap1 and regulate its degradation via clearance of the SQSTM1–Keap1 complex by the ubiquitin–proteasome system, which is triggered by binding of the ubiquitin-associated domain (UBA) of SQSTM1. Upon oxidative stress, the escape of Nrf2 from Keap1 also contributes to the formation of LC3–SQSTM1–Keap1 aggregates (Zhang et al., 2021). In addition, Nrf2 regulates *SQSTM1* gene expression (Ho and Gorski, 2019). Overall, an SQSTM1–Keap1–Nrf2 positive-feedback loop (Zhang et al., 2021) represents the mutual regulatory relationship between the Nrf2 antioxidant system and autophagy (Figure 3).

### 2.2 Transcription factor EB

Transcription factor EB (TFEB), a basic helix-loop-helix-leucine-zipper transcription factor, belongs to the MiT family (Napolitano and Ballabio, 2016). TFEB promotes the expression of a tandem of genes involved in autophagic and lysosomal biogenesis and function (Franco-Juárez et al., 2022; Tan et al., 2022). Thus, TFEB, as a potential therapeutic target, has been associated with many diseases, such as lysosomal storage diseases, neurodegenerative diseases, and cancer (Bajaj et al., 2019; Tan et al., 2022). TFEB agonist treatment has therapeutic effects on several animal models, including Batten disease (Palmieri et al., 2017), NPC disease (Argüello et al., 2021), and Pompe disease (Spampanato et al., 2013).

Overexpression of TFEB leads to degradation of bulk autophagic substrates such as damaged mitochondria (Nezich et al., 2015), long-lived protein aggregates (Martini-Stoica et al., 2016), and lipid droplets (Settembre et al., 2013), indicating the crucial role of TFEB in organelle-specific autophagy, such as mitophagy and lipophagy (Settembre et al., 2013; Nezich et al., 2015). Importantly, TFEB has no effect on the basal transcriptional levels of downstream target genes but rather regulates their transcriptional levels in response to stimuli. Under normal conditions, TFEB is phosphorylated by mTORC1 and exists mainly in an inactive state in the cytosol (Martina and Puertollano, 2013). Upon stimuli such as starvation or oxidative stress, TFEB is dephosphorylated, subsequently translocates to the nucleus, and promotes the activation of its target genes (Sancak et al., 2010; Medina et al., 2015).

Interestingly, Bo Pan et al., 2020 have delineated a relationship between SQSTM1 and TFEB. They proposed that the protein aggregates formed by SQSTM1 during systemic proteasome inhibition sequester mTOR and prevent its TFEB phosphorylation. Furthermore, SQSTM1 is capable of interacting with Raptor, a part of mTORC1, and colocalizing with Rag GTPases at the lysosomal membrane, hence being responsible for the activation of mTORC1 (Duran et al., 2011). SQSTM1 can also recruit the tumor necrosis factor receptor-associated factor 6 in an amino acid-dependent manner and activate mTOR as well (Linares et al., 2013). Notably, *Nrf2/NFE2L2* is identified as a target gene of TFEB; thus, TFEB regulates redox homeostasis via targeting Nrf2 (Li et al., 2021b). Therefore, a network forms that connects Nrf2, SQSTM1, TFEB, and mTOR (Figure 3).

### 2.3 Other non-canonical bridging regulatory factors

Other non-canonical autophagy regulatory proteins like Sestrin2 (Bae et al., 2013) and tripartite motif-containing protein 16 (TRIM16) (Kimura et al., 2015) also participate in the regulation of Nrf2 and mTOR via direct or indirect interactions, providing novel targets for pondering the connection between autophagy and the Nrf2 antioxidant pathway.

#### 2.3.1 Sestrin2

Sestrin2, a highly conserved protein belonging to the Sestrin family, is involved in multiple biological functions, including regulating redox reactions, metabolic homeostasis, and the aging process (Lee et al., 2013; Gong et al., 2021). Sestrin2 has been proposed to be the therapeutic target for various diseases, such as cardiac diseases, respiratory diseases, and non-alcoholic fatty liver disease (Ren D. et al., 2020; Sun et al., 2020; Wu D. et al., 2021). Sestrin2 has been reported to protect insulin-resistant cells by activating the AMPK pathway or upregulating the mTOR pathway (Gong et al., 2021), thus promoting a beneficial effect on diabetes (Tian et al., 2022).

Studies have shown that Sestrin2 overexpression can induce autophagic degradation of Keap1, leading to the upregulation of Nrf2 activity (Bae et al., 2013). In addition, this process requires the phosphorylation of the UBA domain of SQSTM1 at Ser409 (Polito et al., 2014; Rhee and Bae, 2015), resulting in the degradation of LC3–SQSTM1–Keap1 aggregates by selective autophagy.

Sestrin2 regulates autophagy mainly through its intervention with mTORC1 signaling (Wolfson et al., 2016). Upon stress, Sestrin2 activates AMPK, which promotes the phosphorylation of tuberous sclerosis complex 2, leading to mTORC1 inhibition (Wolfson et al., 2016). Sestrin2 can also regulate mTORC1 through GAP activity toward the Rags (n) 1/2 complex (Parmigiani et al., 2014; Rhee and Bae, 2015). GATOR1 inhibits mTORC1 signaling, while GATOR2 positively regulates mTORC1 and inhibits GATOR1 by forming a complex at the lysosomal membrane (Bar-Peled et al., 2013). Upon amino acid starvation, Sestrin2 interacts with GATOR2, thus impeding its interaction with GATOR1, which results in the inactivation of mTORC1 (Chantranupong et al., 2014).

#### 2.3.2 TRIM16

TRIM16 has been identified as a regulator of oxidative-stress-responsive proteins (Ren X. et al., 2020) and is associated with tumor suppression (Kim et al., 2016), cell motility (Huo et al., 2015; Li et al., 2016), apoptosis (Kim et al., 2016), and autophagy (Chauhan et al., 2016; New and Thomas, 2019). TRIM16 deficiency significantly exacerbates cardiomyocyte hypertrophy, while overexpression of TRIM16 inhibits cardiac hypertrophy, suggesting that a TRIM16 inhibitor could be a novel inhibitor of pathological heart hypertrophy and heart failure (Liu J. et al., 2022). Jena et al. (2018) have unveiled the latent significance of TRIM16 in protecting cells from oxidative stress-induced cytotoxicity by interacting with Nrf2, Keap1, and SQSTM1. TRIM16, as an E3 ligase, promotes the SQSTM1-induced autophagic degradation of Keap1 and regulates Nrf2 activity (Jena et al., 2018).

TRIM16 associates with ULK1 and Beclin 1 to engage in autophagy (Chauhan et al., 2016). Furthermore, TRIM16 forms a protein complex with endogenous DEP domain-containing mTOR-interacting proteins, a mTOR inhibitor, and Rag B and Rag D factors, which are responsible for mTOR recruitment to the lysosome (Jena et al., 2018). This TRIM16 complex also interacts with TFEB and calcineurin (Chauhan et al., 2016) (the phosphatase that dephosphorylates TFEB (Medina et al., 2015)). Accumulating evidence illuminates the scaffolding role of TRIM16 in autophagy regulation. Interestingly, both Sestrin2 and TRIM16 are positively regulated by Nrf2 at the transcriptional level (Shin et al., 2012; Jena et al., 2018; Jena et al., 2019). Hence, SQSTM1, Sestrin2, TRIM16, and Nrf2 can also form a complex feedback loop (Figure 3).

### 2.4 Ions and miRNAs

#### 2.4.1 Calcium

Ca^2+^ is the most widespread intracellular messenger (Glaser et al., 2019) whose role in autophagy has been studied extensively. For example, starvation induces lysosomal Ca^2+^ release through the TRPML1 channel, which activates TFEB by calcineurin and promotes autophagy (Medina et al., 2015). Meanwhile, the calcium/calmodulin-dependent protein kinase (CaMK) family, as the major Ca^2+^ signal transducer, plays a crucial role in a variety of autophagy-related signaling pathways, including Ca^2+^-CaMKKβ-AMPK (Woods et al., 2005)-induced autophagy (Kim et al., 2011; Hu et al., 2019). Furthermore, Ca^2+^ channels in various organelles, such as the lysosome or endoplasmic reticulum, have effects on autophagy (Decuypere et al., 2011a; Decuypere et al., 2011b), but this remains controversial.

A recent study has revealed that TRPV1-evoked Ca^2+^ influx promotes CaMKII phosphorylation and subsequently promotes Nrf2 nuclear translocation (Lv et al., 2021). In addition, the Ser558 site in the Neh1 domain of Nrf2 has been identified as a direct phosphorylation site of AMPK (Joo et al., 2016), which results in Nrf2 activation via the Ca^2+^-CaMKKβ-AMPK pathway. Moreover, Ca^2+^-dependent protein kinase C can phosphorylate the Ser40 site in the Neh2 domain of Nrf2 (Niture et al., 2014), thereby activating Nrf2.

#### 2.4.2 miRNAs

At post-transcriptional level, microRNAs (miRNAs) have been reported to impact both the regulation of autophagy and Nrf2 antioxidant signaling. miR-144 is the first miRNA identified as an Nrf2 negative regulator (Sangokoya et al., 2010), and miR-144 can also promote autophagy (Chen et al., 2015). Conversely, Nrf2 can upregulate miR-129-3p, which inhibits mTOR and leads to the initiation of autophagy (Sun et al., 2019). In addition, a study investigated miR-93 and Nrf2 and demonstrated that miR-93 can regulate the mRNA and protein levels of Nrf2 (Singh et al., 2013), while it can target Atg16L to affect autophagy in breast cancer cells (Lu et al., 2014).

## 3 Conclusion

In summary, examining the crosstalk between Nrf2 antioxidant signaling and autophagy provides insights into how they are interconnected and the proteins that mediate their communication. In the aspect of autophagy and the Nrf2-Keap1 antioxidant pathway, SQSTM1, TFEB, Sestrin2, and TRIM16 are all involved in different conditions and act as the scaffolds connecting the two pathways (Table 1). Moreover, studies also reveal that intricate feedback loops form between these bridging proteins. These factors are potential therapeutic targets for diseases with both autophagy dysfunction and oxidative stress. However, since these regulatory proteins seem intricately entangled, potential side effects in practical scenarios should also be taken into consideration. Nevertheless, further studies on understanding the complex crosstalk between autophagy and antioxidant pathways are yet to be conducted.

**TABLE 1:** List of key bridging factors regulating both autophagy and Nrf2 pathway.

Bridging factor	Regulation of autophagy	Regulation of Nrf2
SQSTM1	Regulates mTOR	Binds to Keap1
TFEB	Regulates autophagy-related genes, including *SQSTM1*, *TRPML1*, and *ULK1*	Regulates expression of SQSTM1 and Nrf2
Sestrin2	Regulates mTOR and AMPK	Promotes phosphorylation of SQSTM1 to bind to Keap1
TRIM16	Reacts with ULK1, combines with mTOR, and interacts with TFEB and calcineurin	Promotes SQSTM1-induced autophagic degradation of Keap1
Ca^2+^	Activates TFEB by calcineurin and regulates mTOR by Ca^2+^-CaMKKβ-AMPK	Promotes Nrf2 phosphorylation by Ca^2+^-CaMKKβ-AMPK-Nrf2, Ca^2+^-CaMKII-Nrf2, and Ca^2+^-PKC-Nrf2 pathways

## Figures and Tables

**Table:** 

**AMPK**	AMP-activated protein kinase
**ARE**	antioxidant response element
**Atg**	autophagy-related protein
**CaMK**	calcium/calmodulin-dependent protein kinase
**Cys**	cysteine
**FoxO**	forkhead Box O
**GATOR**	GAP activity toward Rags
**HMOX1**	heme oxygenase-1
**H**_**2**_ **O**_**2**_	hydrogen peroxide
**Keap1**	Kelch-like ECH-associated protein 1
**KIR**	Keap1-interacting region
**LC3**	light chain 3
**LIR**	LC3-interacting region
**mTOR**	mechanistic target of rapamycin
**NAC**	N-acetylcysteine
**Neh**	Nrf2-ECH homology
**NF-κB**	nuclear factor kappa-B
**NPC**	Niemann–Pick type C
**Nrf2**	nuclear factor erythroid 2-related factor 2
O_2_^−^	superoxide
**PD**	Parkinson’s disease
**PE**	phosphatidyl ethanolamine
**PEX**	peroxisomal biogenesis factor
**ROS**	reactive oxygen species
**Ser**	Serine
**sMaf**	small musculoaponeurotic fibrosarcoma
**SQSTM1**	sequestosome1
**TFEB**	transcription factor EB
**TRIM16**	tripartite motif-containing protein 16
**UBA**	ubiquitin-associated domain
**ULK1**	UNC-51-like kinase 1
